# Fungal Communities Within Pitaya Fruit Peel Shift During Ripening and Early Canker Onset

**DOI:** 10.3390/microorganisms14071441

**Published:** 2026-06-30

**Authors:** Ziting Yao, Yanling Zhao, Lianke Zhu, Guining Zhu, Chengwu Zou

**Affiliations:** 1 Plant Protection Research Institute, Guangxi Academy of Agricultural Sciences, Nanning 530007, China; fungiyanlingz@163.com (Y.Z.); zhukeke1016@163.com (L.Z.); zgn@gxaas.net (G.Z.); 2 Key Laboratory of Green Prevention and Control on Fruits and Vegetables in South China, Ministry of Agriculture and Rural Affairs, Nanning 530007, China; 3 Guangxi Key Laboratory of Biology for Crop Diseases and Insect Pests, Nanning 530007, China; 4 College of Agriculture, Guangxi University, Nanning 530004, China; 5 National Demonstration Center for Experimental Plant Science Education, Nanning 530004, China

**Keywords:** pitaya microbiome, community assembly, co-occurrence network, trophic guilds, fruit maturity, canker disease

## Abstract

Canker is a major fungal disease that causes substantial yield losses in pitaya (*Selenicereus monacanthus* (Lemaire) D.R.Hunt, syn. *Hylocereus polyrhizus* (F.A.C. Weber) Britton and Rose; red-fleshed pitaya). However, how fruit ripening and pathogenesis interactively shape fungal communities in fruit peels remains unclear. Here, we investigated the diversity, assembly mechanisms, co-occurrence networks, and functional guilds of fungal communities in healthy and diseased fruit peels at immature (green) and mature (red) stages of ‘Jindu No. 1’ pitaya using ITS1 amplicon sequencing. Our results revealed that fruit maturity exerted stronger effects on fungal community structure than disease status, with ripening reducing diversity and increasing dominance. Notably, disease-induced stage-dependent responses: immature communities shifted from stochastic to deterministic assembly under pathogen selection, whereas mature communities maintained stochastic processes despite infection. Co-occurrence network analysis revealed that healthy mature peels formed highly complex, cooperative networks with dense positive interactions, while healthy immature peels exhibited fragmented, modular structures vulnerable to invasion. Diseased immature peels displayed intermediate network topology, and diseased mature peels showed disrupted connectivity. Functionally, healthy fruits maintained balanced pathotroph-saprotroph-symbiotroph guilds, whereas diseased fruits exhibited higher relative abundance of pathotrophs and saprotrophs, reflecting a shift from symbiotic nutrient cycling toward necrotrophic pathogenicity and decomposition. These findings challenge the single-pathogen paradigm by revealing canker as an ecological process involving community-wide restructuring. They provide a theoretical basis for stage-specific microbiome-targeted disease management in tropical fruits, emphasizing the preservation of stochastic assembly and cooperative network structures to enhance disease resistance.

## 1. Introduction

Pitaya (*Selenicereus monacanthus* (*Lemaire*) D.R.Hunt, also classified as *Hylocereus polyrhizus* (F.A.C. Weber) Britton and Rose; red-fleshed pitaya or red dragon fruit), has emerged as a significant tropical fruit crop with its commercial cultivation expanding globally. This rapid expansion reflects growing market demand and economic value, yet also exposes the crop to potentially significant disease threats in new cultivation regions. However, the fungal canker disease causes substantial yield losses in this crop, threatening the sustainability of the pitaya industry. Pitaya stem, and fruit canker is primarily caused by the fungi, mainly *Neoscytalidium dimidiatum* (Penz.) Crous and Slippers, *Neoscytalidium hylocereum* Dy et al., and *Lasiodiplodia theobromae* (Pat.) Griffon and Maubl. This disease has spread globally to major pitaya-producing regions, including China, Malaysia, Israel, Mexico, and the US, causing severe economic losses. In China, the world’s largest pitaya producer, the disease affects over 50% of plantations in high-incidence areas, with annual economic losses exceeding USD 27 million [1]. It typically results in grayish-white or brown spots on the stem and peel and causes “scabby skin fruit” in severe cases, compromising fruit quality and marketability [2,3,4,5]. Conventionally, researchers focused on pathogen identification, epidemiological monitoring, and chemical/biological control strategies. However, the microbial communities within the stem or fruit peel of pitaya and their potential ecological roles during fungal canker pathogenesis have not been explored [6,7,8].

In the plant microbiome, the common and functionally critical components are the fungi, which form complex symbiotic relationships with their hosts. These fungal symbionts promote nutrient absorption, enhance stress tolerance, and synthesize bioactive secondary metabolites, significantly improving the environmental adaptability of host plants [9,10,11]. Furthermore, these endophytes critically mediate disease outcomes, with beneficial species suppressing pathogen invasion through antagonistic interactions, whereas opportunistic taxa may exacerbate disease severity when the microbial balance is disrupted. Specifically, the fungal community colonizing the peel tissue serves as a crucial defensive barrier against pathogen invasion. These fungal communities function through ecological niche occupation, resource competition, and host resistance induction [12,13]. During infection, pathogens invading plants disrupt the homeostasis of indigenous microbial communities through various mechanisms, such as competing for resources with established host microorganisms, modifying the physicochemical properties of plant microenvironments, and modulating the defense responses of host plants. Research has shown that pathogens disrupt the ecological balance of the microbial community on fruit peels, potentially eradicating sensitive symbiotic bacteria and promoting saprotrophic fungi and other opportunistic colonizers that exploit vacant niches in the infected necrotic tissue [14]. However, the diversity, assembly, and spatiotemporal dynamics of fungi associated with fruit peels during fruit ripening, especially their role in regulating responses to fruit disease, while studied in *Malus domestica* (apple) and *Capsicum annuum* (bell pepper), remain unclear in pitaya [15,16].

The fruit ripening stage represents a critical window when the susceptibility of fruits to pathogens typically reaches its peak [17,18]. Ripening involves a profound physiological shift that alters the microenvironment of the fruit and drives the succession of its microbial communities. Throughout this process, the fruit undergoes a series of internal changes, including degradation of chlorophyll, accumulation of carotenoids, remodeling of epidermal wax, and an increase in soluble sugars. These changes collectively modify the availability of nutritional resources and the suitability of the physical habitat for fungal colonization within the fruit tissues. Research in various species, such as grape and *Rubus chingii* Hu, has demonstrated that fruit maturity significantly influences the diversity and composition of endophytic fungal communities on or within the fruit [19,20]. The ripening of grapes significantly altered the fungal microbiome on the fruit surface: species richness peaked during the middle stage of development and then declined, while the overall diversity and evenness of the community slightly decreased during the later stage of ripening. In the immature stage, the dominant fungal genera included *Aspergillus*, *Malassezia*, *Metschnikowia*, and *Udeniomyces*; as the fruit ripens, the dominant fungal communities shifted to *Erysiphe*, *Cryptococcus*, *Vishniacozyma*, and *Cladosporium* [19]. The richness and diversity of endophytic fungi in the fruits of *R*. *chingii* continued to increase as the fruit ripened. Although *Cladosporium* dominated at all stages, the abundance of many other genera underwent significant changes. For example, *Udeniomyces* had the highest abundance in the early stage (light green stage) and then had lower relative abundance; whereas genera such as *Epicoccum*, *Taphrina*, and *Eremothecium* were significantly enriched in the fully ripe stage (red stage) [20]. These studies have indicated that as fruit matures, the diversity of fungal taxa that rely on specific carbon sources declines, while the abundance of dominant taxa rises. However, whether ripening (transition fromimmature to mature peel) in pitaya orchestrates and drives the succession of fruit-associated fungal communities remains unknown.

Studies have demonstrated that the succession of microbial communities on the fruit surface during fruit maturation exhibits distinct regularities [21]. Generally, microbial communities shift from a symbiotic-dominated microbiome in unripe fruits to a decay-promoting composition in ripe fruits, aligning with the increase in sugar content and the deterioration of the epidermal barrier. These studies indicate that fruits at different developmental stages share certain stable microbial taxa, suggesting that mature fruits retain microbial communities from the juvenile stage. These microbial communities have been shown to influence the risk of disease occurrence after fruit ripening [21]. However, the succession patterns of fungal communities during pitaya ripening and their association with diseases such as canker, while documented in peach and apple, where ripening increases susceptibility to pathogens like *Monilinia laxa* (Aderh. and Ruhland) Honey and *Penicillium expansum* Link, remain poorly understood [22]. Therefore, the present study aimed to elucidate the fungal communities underlying canker disease occurrence across different ripening stages, providing novel insights into disease-microbe interactions and a foundation for developing stage-specific management strategies. Considering the fact that both immature and mature fruits are susceptible to canker disease, we postulated that the response patterns of fungal communities to canker differ across fruits of different maturity stages. To test this hypothesis, we analyzed healthy and diseased peels from pitaya fruits at the immature and mature peel stages, performed high-throughput amplicon sequencing, and compared the composition, diversity, and co-occurrence networks of fungal communities.

## 2. Materials and Methods

### 2.1. Study Site and Experimental Design

**Figure 1:**
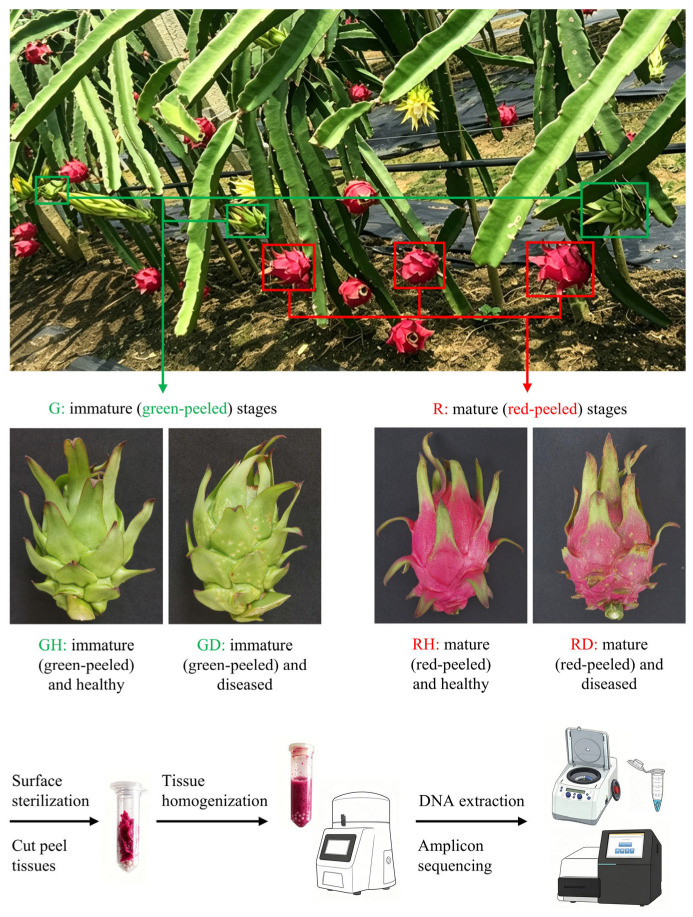
Experimental design and sample processing workflow. Representative photographs of pitaya fruits from the ‘Jindu No. 1’ cultivar in an orchard in Nanning, Guangxi, illustrating the four experimental groups: immature (green-peeled) and healthy (GH), immature (green-peeled) and diseased (GD), mature (red-peeled) and healthy (RH), and mature (red-peeled) and diseased (RD). The lower panel shows the laboratory workflow for fruit-associated fungal community profiling, from surface sterilization of peel tissues to paired-end amplicon sequencing of the fungal ITS1 region.

The pitaya cultivar “Jindu No. 1” cultivated at the scientific research base of the Guangxi Academy of Agricultural Sciences in Nanning, Guangxi (108°12′56″ E, 23°14′44″ N) was used in this study. The plantation was established in a cement column planting system with a row spacing of 2.5 m and a plant spacing of 2.0 m. Based on historical observations from 2020 to 2022, canker disease occurs spontaneously and randomly in the orchard, with peak incidence in July and August. Accordingly, samples were collected on 23 June 2023, prior to the seasonal disease peak. Fruits were collected from two developmental stages: 25 days post-flowering (DPF; immature, green-peeled, at the end of fruit expansion) and 35 DPF (mature, red-peeled, ripe). By selecting cohorts with synchronized anthesis dates, we ensured that immature fruits (green-peeled) had reached full size (post-expansion) rather than small, developing fruits, while mature fruits (red-peeled) represented the ripe stage from an earlier flowering batch. Prior to sampling, fruits were assessed for canker severity using a 0-to-9 disease rating scale, where 0 = no symptoms, 1 = necrotic lesions covering ≤ 5% of the peel, 3 = necrotic lesions covering 6~10% of the peel, 5 = necrotic lesions covering 11~25% of the peel, 7 = necrotic lesions covering 26~50% of the peel, 9 = necrotic lesions covering ≥ 51% of the peel. The fruit samples collected at each stage were categorized by canker severity as follows: healthy samples with scale 0 (no visible lesions) and early-stage diseased samples with scale 3 (6~10% surface lesion coverage). Only these two severity levels were included in the subsequent microbiome analysis (Figure 1). Thus, four experimental groups were analyzed in this study, with eight replicates per group: immature (green-peeled) and healthy (GH), immature (green-peeled) and diseased (GD), mature (red-peeled) and healthy (RH), and mature (red-peeled) and diseased (RD) groups. The samples collected were placed in sterile sampling bags and immediately transported to the laboratory. No fungicides were applied to the plants throughout fruit development and sampling.

### 2.2. Sample Processing and DNA Extraction

The surface of the sampled fruits was rinsed with sterile distilled water, disinfected with 75% ethanol (three sequential washes), and air-dried in a laminar flow hood in the laboratory. Then, the peel tissues (exocarp and mesocarp) were excised using sterilized scalpels, cut into 1 cm^2^ pieces, and snap-frozen in liquid nitrogen. Microbial genomic DNA was then extracted from 2 g of peel tissue per sample using the E.Z.N.A.^®^ Soil DNA Kit (Omega Bio-Tek, Norcross, GA, USA), selected for its bead-beating lysis capability that effectively disrupts fungal cell walls and its established efficacy in plant tissue and fruit microbiome studies [23]. The quality of the extracted DNA was assessed via 1% agarose gel electrophoresis, and its concentration was assessed using a NanoDrop 2000 spectrophotometer (Thermo Fisher Scientific, Wilmington, DE, USA).

### 2.3. PCR Amplification and Sequencing

The internal transcribed spacer 1 (ITS1) region of fungal DNA was amplified using the primers ITS1F (5′-CTTGGTCATTTAGAGGAAGTAA-3′) and ITS2 (5′-GCTGCGTTCTTCATCGATGC-3′) [24]. The 20 μL mixture used for PCR contained 4.0 μL of 5× TransStart FastPfu buffer, 2.0 μL of 2.5 mM dNTPs, 0.8 μL of each primer, 0.4 μL of TransStart FastPfu DNA polymerase (TransGen Biotech, Beijing, China), and 10 ng of template DNA. The reaction was carried out under the following thermal cycling conditions: an initial denaturation at 95 °C for 3 min; 30 amplification cycles at 95 °C for 30 s, 55 °C for 30 s, 72 °C for 30 s; and a final extension at 72 °C for 10 min. PCR products from individual samples (n = 8 biological replicates per group) were purified using the AxyPrep DNA Gel Extraction Kit (Axygen, California, CA, USA) and quantified with a Qubit 4.0 Fluorometer (Thermo Fisher Scientific, Waltham, MA, USA). Finally, libraries were constructed using these amplicons with the TruSeq Nano DNA LT Library Prep Kit (Illumina, San Diego, CA, USA) and sequenced on an Illumina MiSeq platform (PE300; Illumina, San Diego, CA, USA) to generate paired-end reads.

### 2.4. Bioinformatics and Statistical Analyses

The raw sequencing reads were processed using fastp (v0.19.6) for primer removal and quality filtering, followed by paired-end assembly using FLASH (v1.2.7). Amplicon sequence variants (ASVs) were inferred using the DADA2 pipeline (QIIME2 plugin), which preserves single-nucleotide resolution and avoids ecological signal loss associated with arbitrary operational taxonomic unit (OTU) clustering thresholds [25,26,27]. Stringent DADA2 parameters were applied to minimize spurious ASV inflation from intra-genomic ITS variation [28]. The fungal sequence counts of each sample were normalized based on the minimum sequence count of all samples (n = 32), and the normalized sequences were subjected to further analysis. The representative sequences of ASVs were aligned against the Unite database v8.0 for species annotation; the process generated the specific composition of fruit-associated fungal communities at various taxonomic levels in each sample. Additionally, Venn diagram analysis was conducted using the jvenn online server (http://jvenn.toulouse.inra.fr/app/example.html) (accessed on 9 February 2026).

Alpha diversity metrics, namely Chao1, Shannon, and Simpson indices, were calculated in the Mothur (v1.30) software, and the differences in these indices among the four experimental groups (GH, GD, RH, and RD) were assessed using Kruskal-Wallis tests with False Discovery Rate (FDR) corrections, followed by a pairwise Dunn’s test. Beta diversity was assessed using Bray-Curtis dissimilarity, and differences in beta diversity were visualized via Principal Coordinates Analysis (PCoA). Additionally, an analysis of similarities (ANOSIM) with 999 permutations was conducted to test for differences in community structure among the four groups. PERMANOVA (999 permutations) was performed using the vegan package in R (v3.3.1) to assess the significance of variance in fungal community structure among the four experimental groups (GH, GD, RH, and RD). Indicator species analysis was performed using the online LEfSe (Linear Discriminant Analysis (LDA) Effect Size) tool available at https://huttenhower.sph.harvard.edu/galaxy/ (accessed on 2 March 2026) with default parameters, and the taxa with LDA score > 4.0, *p* < 0.05 from phylum to genus levels were identified as discriminative between healthy and diseased groups within each ripening stage.

The ecological assembly processes of fruit-associated fungal communities were quantified using the normalized stochasticity ratio (NST) calculated with the NST package (v3.1.10). An NST value > 0.5 indicated that stochastic processes (dispersal limitation and drift) dominated community assembly, while a value < 0.5 indicated dominance by deterministic processes (selection). Further, co-occurrence networks were constructed using Spearman’s correlations (|ρ| > 0.6, FDR-adjusted *p* < 0.05) using the Networkx package (v1.11) in Python-2.7. The network topological parameters, such as modularity, clustering coefficient, betweenness centrality, and average path length, were calculated using Gephi (v0.10.1). Functional analysis of fruit-associated fungal communities was predicted using the FUNGuild online tool available at http://www.funguild.org/ (accessed on 4 March 2026). The relative abundances of each functional guild were calculated by summing the abundances of ASVs assigned to that category. Finally, the differential abundance of these guilds was determined using Kruskal-Wallis tests with FDR corrections, followed by a pairwise Dunn’s test.

## 3. Results

### 3.1. Fungal Community Diversity and Taxonomic Composition Across Ripening Stages and Disease Statuses

A total of 1,953,170 ITS sequences obtained from 32 samples of pitaya fruit were analyzed. After quality filtration, a total of 1,716,433 high-quality ITS reads were obtained, with an average of 53,638 ± 8170 reads per sample. These reads were rarefied to the lowest sequencing depth (31,260 sequences per sample) and assigned to 1185 ASVs (98 ± 26 ASVs per sample). All subsequent alpha- and beta-diversity analyses were performed on the complete set of 1185 ASVs (100% coverage). A Venn diagram based on these data displayed that 78 fungal ASVs were shared across the four experimental groups, while a few were group-specific. Notably, 246 ASVs were specific to immature healthy (GH) fruits, 296 to immature diseased (GD) fruits, 219 to mature healthy (RH) fruits, and 153 to mature diseased (RD) fruits (Figure S1). Of the 1185 ASVs, 87.2% were assigned to phylum, 82.0% to class, 81.6% to order, 79.1% to family, and 72.5% to genus. ASVs that could not be classified at the phylum level accounted for 1.3% of total sequences.

**Figure 2:**
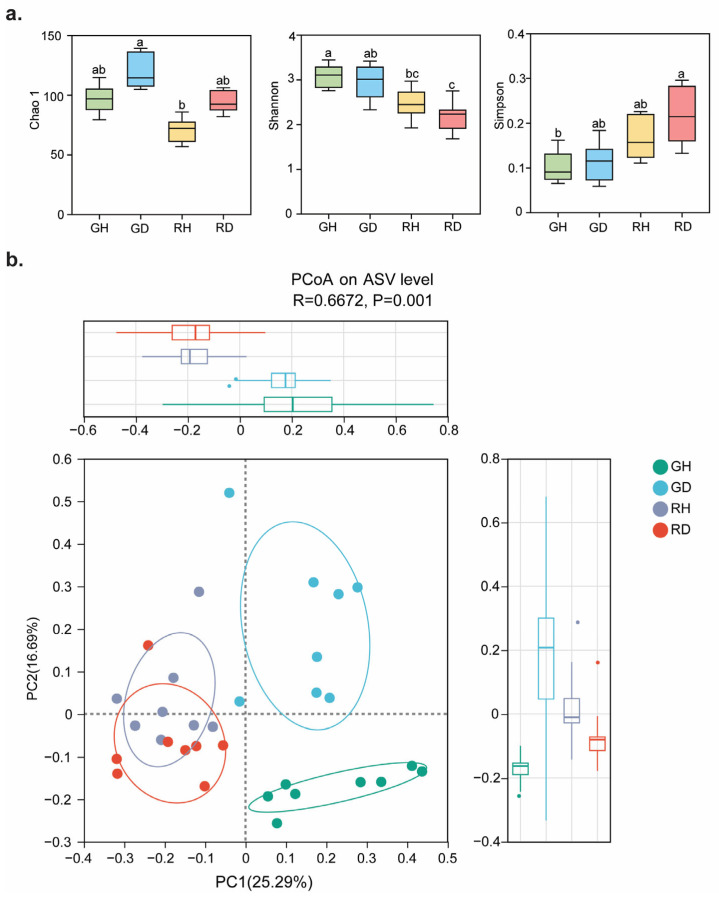
Alpha diversity and Beta diversity at the ASV level of fruit-associated fungal communities. (**a**) Alpha diversity (Chao1 index, Shannon’s index and Simpson’s index) of fungal communities among the four groups. Different lowercase letters above the bars indicate statistically significant differences according to a pairwise Dunn’s test (*p* < 0.05, Kruskal-Wallis tests with FDR corrections). (**b**) PCoA of fungal communities at the ASV level among the four groups. ANOSIM was conducted to test for differences in community composition resulting from ripeness and disease status. Circles and ellipses are colored in groups. The ellipse represents the standard deviation between the axes and the centroids of the groups. The green, blue, purple and red circles respectively represent the peel samples of the four groups. GH: immature and healthy; GD: immature and diseased; RH: mature and healthy; RD: mature and diseased.

Alpha diversity analysis revealed significant differences in the diversity and richness of fungal communities among the four groups (Figure 2a). The Chao1 index was significantly higher in GD than in RH (115.456 vs. 87.365; *p* < 0.05). The Shannon index was highest in GH, significantly exceeding both RH and RD (3.078 vs. 2.463 and 2.192; *p* < 0.05). The Simpson index was highest in RD, significantly exceeding GH (0.216 vs. 0.103; *p* < 0.05).

### 3.2. Beta Diversity and PERMANOVA Revealed That Ripening Stage Drives Fungal Community Structure More Strongly than Disease Status

Beta diversity analysis also revealed that both ripening stage and disease status significantly influenced the clustering patterns of the fruit-associated fungal community. PCoA based on Bray-Curtis dissimilarity displayed clear separation of samples along the first two principal coordinate axes, which accounted for 31.98% of the cumulative variance in the fungal community composition (Figure 2b). PERMANOVA revealed that both ripening stage (R^2^ = 0.2066, *p* < 0.001) and disease status (R^2^ = 0.1354, *p* < 0.001) significantly structured the fungal communities, with ripening stage contributing more to the variation than disease status (Table 1). The interaction between the two factors explained an additional 12.8% of the variation (R^2^ = 0.1280, *p* < 0.001), and the full model collectively accounted for 47.0% of the total variation (R^2^ = 0.470). Notably, even with only ~10% lesion coverage, disease status explained 13.54% of the community variation, whereas ripening stage explained 20.66%.

### 3.3. Taxonomic Composition Shifted Toward Ascomycota Dominance in Diseased Immature Fruits

**Figure 3:**
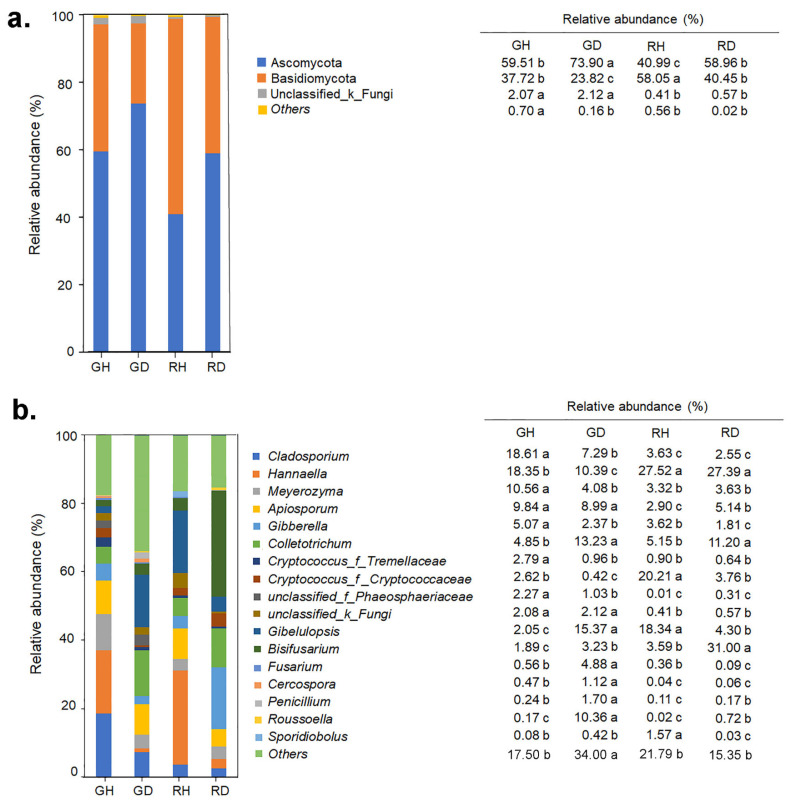
Relative abundance of dominant (relative abundance > 1%) phyla (**a**) and genera (**b**) in fruit-associated fungal communities in the four groups. Different lowercase letters in the table indicate statistically significant differences according to a pairwise Dunn’s test (*p* < 0.05, Kruskal-Wallis tests with FDR corrections). GH: immature and healthy; GD: immature and diseased; RH: mature and healthy; RD: mature and diseased.

Subsequent analyses revealed clear differences in fungal taxonomic composition at both phylum and genus levels among the four experimental groups (GH, GD, RH, and RD). At the phylum level, Ascomycota (28.55–94.92%) and Basidiomycota (4.83–71.08%) dominated all samples. Ascomycota had significantly higher relative abundance in GD (73.90%, *p* < 0.05), whereas Basidiomycota showed the highest relative abundance in RH (58.05%) and the lowest in GD (23.82%) (Figure 3a). At the genus level, *Hannaella* was significantly more abundant in mature peel groups (RH and RD) than in immature peel groups (GH and GD). Conversely, *Cladosporium*, *Apiotrichum*, and unclassified*_Phaeosphaeriaceae* had higher relative abundance in immature peel groups. Further comparison of the healthy and diseased fruits at each ripening stage revealed distinct patterns. In the immature groups, GH was characterized by *Cladosporium*, *Meyerozyma*, *Gibberella*, and *Cryptococcus*, whereas GD exhibited significantly higher relative abundance of *Gibellulopsis*, *Colletotrichum*, *Roussoella*, *Fusarium*, and *Penicillium*. In the mature groups, RH maintained a higher relative abundance of *Gibellulopsis* and *Sporidiobolus*, while RD showed higher relative abundance of *Colletotrichum* and *Bisifusarium* (Figure 3b).

### 3.4. Indicator Species Analysis Identified Distinct Fungal Biomarkers for Each Ripening Stage and Health Status

**Figure 4:**
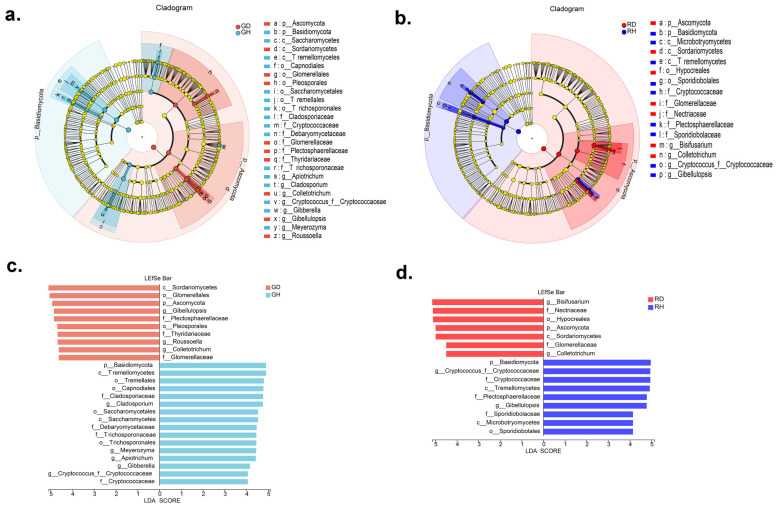
LEfSe analysis of fruit-associated fungal community between healthy and diseased groups at different ripeness stages. Cladograms showing taxonomic representation of significant differences between (**a**) GH and GD, and (**b**) RH and RD. Colored nodes from the inner to the outer circles represent taxa from the phylum to the genus level. Significantly different taxa are marked with distinct colors corresponding to the four groups. Histogram of LDA scores for differentially abundant features between (**c**) GH and GD, and (**d**) RH and RD. A logarithmic LDA score threshold of 4.0 was used to identify discriminative features. GH: immature and healthy; GD: immature and diseased; RH: mature and healthy; RD: mature and diseased.

Indicator species analysis using LEfSe identified 26 differentially abundant taxa encompassing 2 phyla, 3 classes, 6 orders, 7 families, and 8 genera between GH and GD, and 16 discriminative taxa (encompassing 2 phyla, 3 classes, 2 orders, 5 families, and 4 genera) between RH and RD (Figure 4a,b). Ascomycota and Basidiomycota remained the dominant phyla across all four groups, collectively accounting for >95% of the sequences in each sample. No single genus exhibited consistently high abundance across all groups; instead, distinct genera dominated each condition (e.g., *Cryptococcus_f__Cryptococcaceae* in healthy groups versus *Colletotrichum* in diseased groups). Specifically, the top indicator taxa (those with the highest LDA scores > 4.0) were: GH was characterized by *Cladosporium*, *Meyerozyma*, *Apiotrichum*, *Gibberella*, and *Cryptococcus_f__Cryptococcaceae*, while GD was distinguished by *Gibellulopsis*, *Roussoella*, and *Colletotrichum* (Figure 4c). In the fruits with mature peel, *Cryptococcus _f__Cryptococcaceae* and *Gibellulopsis* were indicators of a healthy state (RH), whereas *Bisifusarium* and *Colletotrichum* were indicators of disease incidence (RD) (Figure 4d).

### 3.5. Ecological Assembly Processes Transition from Stochastic to Deterministic in Diseased Immature Fruits Only

**Figure 5:**
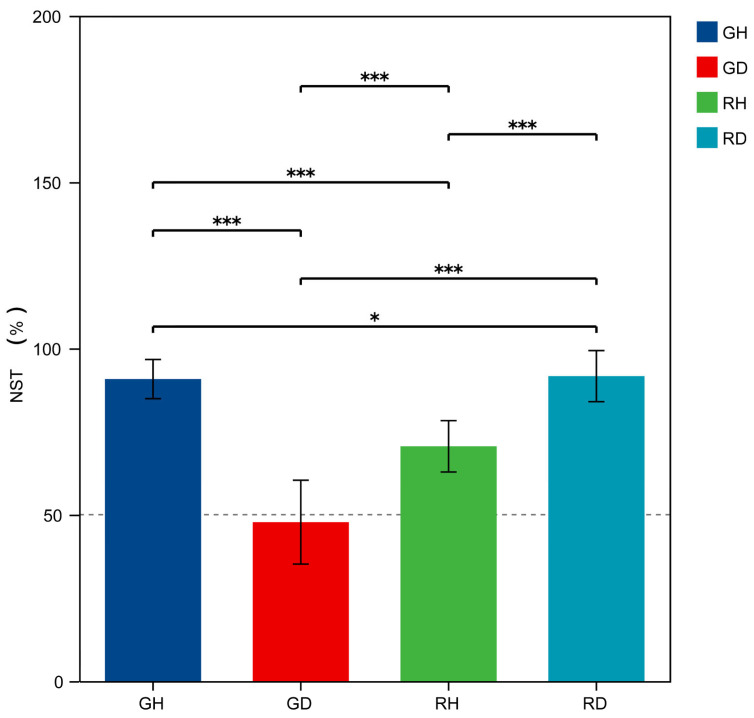
Normalized stochasticity ratio (NST) values for fruit-associated fungal community assembly mechanisms. The four groups are represented by distinct colors, and vertical dashed lines indicate the mean NST value for each group. Wilcoxon test with Holm correction was performed. * *p* ≤ 0.05, *** *p* ≤ 0.001. GH: immature and healthy; GD: immature and diseased; RH: mature and healthy; RD: mature and diseased.

To elucidate the ecological forces governing community assembly, null model analysis based on NST was performed to quantify the relative contributions of stochastic and deterministic processes. In the immature stage, the healthy group (GH) exhibited an NST value of 0.908, whereas the diseased group (GD) showed a marked decrease to 0.477. In contrast, both mature groups (RH and RD) maintained NST values above 0.5, consistent with stochastic dominance regardless of disease status (Figure 5).

### 3.6. Canker Disease Reshapes Fungal Co-Occurrence Network Topology in a Ripening Stage-Dependent Manner

**Figure 6:**
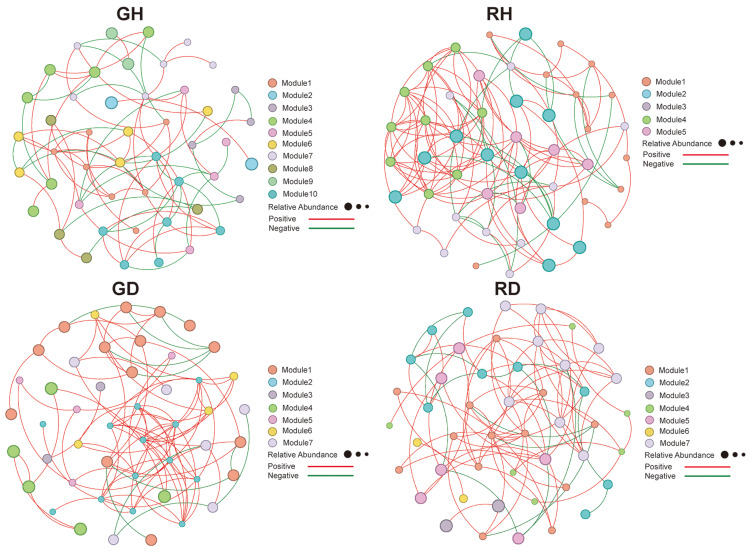
Co-occurrence networks of fruit-associated fungal communities for each group. Each node represents an ASV, with node color indicating the corresponding module class as shown in the legend, and node size reflecting the relative abundance of the ASV. Edges represent correlations between pairs of ASVs, with red edges indicating positive correlations and green edges indicating negative correlations. GH: immature and healthy; GD: immature and diseased; RH: mature and healthy; RD: mature and diseased.

Further, we constructed correlation-based networks to determine the impact of canker disease on microbial co-occurrence patterns (Figure 6). Co-occurrence networks were constructed from ASVs with significant Spearman correlations (|ρ| > 0.6, FDR-adjusted *p* < 0.05), resulting in 47–48 nodes per group (Table 2). To quantify community complexity and interaction patterns, we calculated network topological parameters including connectivity, clustering coefficient, modularity, and average path length (Table 2). Compared to the GD network, the GH network had fewer edges (GH/GD: 79/95), lower betweenness centrality (GH/GD: 0.064/0.083), higher clustering coefficient (GH/GD: 0.387/0.381), and higher average path length (GH/GD: 4.509/4.397), and lower average degree (GH/GD: 3.292/3.958). Meanwhile, compared to the RD network, the RH network had more edges (RH/RD: 118/87), lower betweenness centrality (RH/RD: 0.026/0.048) and average path length (RH/RD: 2.911/3.558), higher clustering coefficient (RH/RD: 0.523/0.398) and average degree (RH/RD: 4.917/3.702). The network of the RH group exhibited a high degree of positive interactions (97 positive edges) among nodes, with the highest network density (0.105) and clustering coefficient (0.523). On the contrary, the network of the GH group displayed the lowest positive interactions (63.29%), the highest modularity (0.673), the largest number of communities (10), and the longest average path length (4.509). Although GD had a high proportion of positive edges (88.42%), its clustering coefficient (0.381) and modularity (0.534) were moderate.

### 3.7. Disease and Ripening Jointly Driven Shifts in Fungal Trophic Modes and Functional Guilds

**Figure 7:**
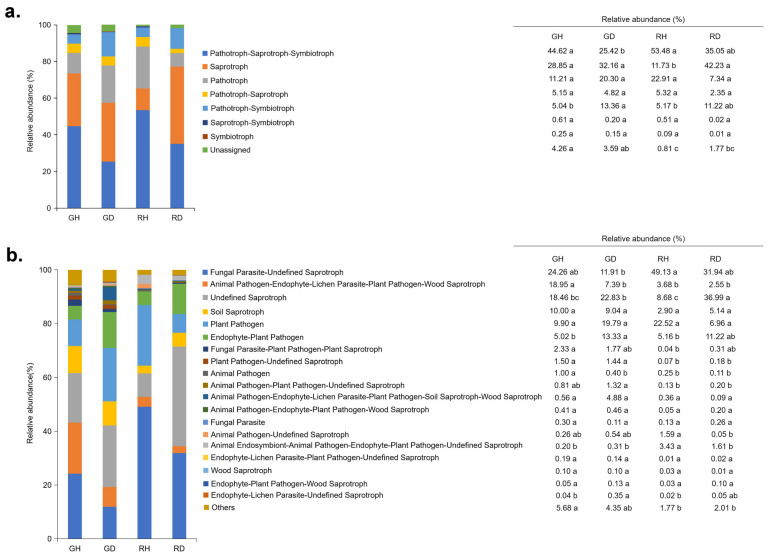
FUNGuild-based functional prediction analysis of fruit-associated fungi, showing significantly different functional modes (**a**) and guilds (**b**) among the four groups. Different lowercase letters in the table indicate statistically significant differences according to a pairwise Dunn’s test (*p* < 0.05, Kruskal-Wallis tests with FDR corrections). GH: immature and healthy; GD: immature and diseased; RH: mature and healthy; RD: mature and diseased.

FUNGuild functional prediction successfully assigned trophic modes and guilds to 889 ASVs (75.0% of the total ASV pool), which collectively accounted for 97.4% of the total sequence abundance. The remaining 296 ASVs (25.0% of ASVs, but only 2.6% of sequences) were either unclassified or designated as unknown saprotrophs. FUNGuild analysis revealed distinct shifts in trophic strategies across ripening stages and disease states. At the primary trophic mode (Figure 7a), the multifunctional pathotroph-saprotroph-symbiotroph modes were the highest in the RH group; the proportion in RH (53.48%) significantly exceeded that in RD (35.05%, *p* < 0.05) and GD (25.42%, *p* < 0.05). Conversely, the pathotrophic mode exhibited significantly higher relative abundance in GD (22.35%) compared to GH (11.21%, *p* < 0.05). The abundance of saprotrophic modes was the highest in RD (42.23%) (*p* < 0.05) but the lowest in GH and RH (26.85% and 11.73%, respectively). The pathotroph-saprotroph modes showed significantly higher relative abundance in GD relative to GH. Meanwhile, at the refined guild level (Figure 7b), plant pathogen and endophyte-plant pathogen guilds had specifically higher relative abundance in GD (*p* < 0.05). In contrast, RH retained significantly higher abundance of fungal parasite-undefined saprotroph guilds than RD (*p* < 0.05). On the other hand, RD showed pronounced higher relative abundance of undefined saprotroph and wood saprotroph guilds. The animal pathogen-plant pathogen-undefined saprotroph complex was significantly more abundant in GH than in diseased groups (*p* < 0.05).

## 4. Discussion

The present study revealed that fruit ripening and canker disease act synergistically as ecological filters, restructuring the fungal communities in pitaya peels [29,30]. At the alpha-diversity level, immature fruits harbored higher fungal richness and diversity than mature fruits, as reflected by elevated Chao1 and Shannon indices in the immature groups compared to the mature groups. We found that ripening contributed the greatest to fungal community variance (R^2^ = 0.2066, *p* < 0.001), while disease status accounted for a substantial proportion of community variance even at early infection stages (~10% lesion coverage; R^2^ = 0.1354, *p* < 0.001). These observations are consistent with the hypersensitivity of fruit microbiomes to pathogen invasion, regardless of ripening stage. Additionally, the significant interaction between the two factors explained an additional 12.8% of the variation (R^2^ = 0.128, *p* < 0.001), indicating that the microbial response to disease was stage-dependent. Collectively, the full model accounted for 47.0% of the total variation in community structure. Specifically, the immature and mature peels exhibited distinct pathways of community restructuring in response to pathogen challenge: immature fruits shifted toward deterministic pathogen-dominated assemblages, whereas mature fruits maintained stochastic assembly but with increased saprotrophic turnover. This finding is consistent with the Anna Karenina principle in disease ecology, which proposes that microbial dysbiosis manifests as excessive pathogen proliferation and an imbalance in host-microbe homeostasis at specific developmental stages [31]. Accordingly, our results suggest that healthy pitaya fruits harbor convergent, stable microbial networks, whereas diseased fruits diverge into distinct stage-specific dysbiotic states—deterministic pathogen invasion in immature fruits versus stochastic saprotrophic decomposition in ripe fruits—suggesting that effective disease management must be tailored to fruit developmental stages rather than applying uniform strategies across ripening phases.

In the immature peels of pitaya fruits afflicted by canker disease, the fruit-associated fungal community exhibited a marked transition from stochastic to deterministic assembly upon pathogen infection (during early infection stages): healthy immature fruits (GH) displayed stochastic dominance (NST = 0.908 > 0.5), whereas diseased immature fruits (GD) shifted to deterministic assembly (NST = 0.477 < 0.5). This signified a profound alteration in ecological mechanisms during the transition from health to disease in immature fruits, overriding the neutral processes (random dispersal and ecological drift) that governed community assembly in healthy fruits. In a healthy state, neutral processes (random dispersal and ecological drift) governed community assembly in the fruit, thereby preserving functional redundancy and species coexistence [32,33]. In contrast, disease exerted a selective filtering influence on the fungal community (NST = 0.477), potentially triggered by pathogen-induced alterations in the microenvironment, such as variations in pH and release of cell wall degradation byproducts, as well as the secretion of antimicrobial compounds. Determinism in the diseased immature samples (GD) may have favored taxa with stress tolerance or rapid necrotic metabolic capacities, effectively suppressing the stochastic mechanisms that sustain diversity in healthy tissues. Comparable deterministic shifts, where environmental filtering under pathogen stress selected for stress-resistant or pathogen-associated taxa, had been documented in other plant-microbe systems, such as tomato rhizosphere and root endosphere bacterial communities affected by bacterial wilt disease (where deterministic processes dominated bacterial assembly) and tobacco stems co-infected with *Ralstonia solanacearum* (Smith 1896) Yabuuchi et al. 1996 and *Neocosmospora falciformis* (Carrión) L. Lombard and Crous 2015, a co-infection that induces deterministic fungal community restructuring [34,35]. Thus, our findings suggest that disease does not merely act as an ancillary perturbing factor but rather emerges as a selective force capable of superseding neutral assembly dynamics, driving the convergent evolution of fungal communities towards a pathogen-predominated state. Practically, this highlights the potential importance of stage-specific disease management: preventing initial pathogen colonization in immature (green-peel) fruits is critical, as once deterministic assembly is established, the community trajectory becomes difficult to reverse through conventional biological control agents that rely on stochastic colonization.

Furthermore, the topology of the co-occurrence networks generated in this study revealed the ecological effects of the transition in the assembly of fruit-associated fungal communities. The healthy mature peel (RH) exhibited a “highly collaborative network” with a high clustering coefficient (0.523), short path length (2.911), and dense positive interactions (97 edges), consistent with a potentially stable and functionally integrated community. In contrast, the healthy immature peel (GH) exhibited a modular and fragmented network organization, with betweenness centrality (0.064) and the longest path length (4.509). This structural pattern is characteristic of subcommunities with weak interactions and limited functional redundancy [36]. Theoretically, the modular structure of this GH network may help slow the spread of biotic disturbances, particularly pathogen invasion; however, the low network connectivity (density: 0.07) and the high proportion of negative edges (36.71%) in these GH fungal networks may reflect loose regulation and fierce competition within this community. Consequently, despite the theoretical protective advantage of modularity, the observed low integration and high antagonism suggest that these immature fruit communities may be vulnerable to pathogen establishment, contrasting sharply with the robust cooperative architecture of mature healthy fruits [28]. On the other hand, the fungal network of diseased immature peel (GD) was in an unstable transitional state. Although the proportion of positive edges was 88.42%, this network had moderate modularity (0.534) and lower density (0.084). Similar transitional network disruptions have been observed in other plant-pathogen systems, such as in chili pepper upper stem affected by *Fusarium*, where deterministic pathogen invasion transiently destabilizes native microbial networks before a stable disease-associated community establishes [37]. These observations raise the possibility that pathogen-driven deterministic assembly may be disrupting the original collaborative network of the fruit-associated fungi, yet a stable disease-associated community has not formed.

Additionally, the distinct functional characteristics of the fruit-associated fungal communities identified by FUNGuild analysis were consistent with the changes observed in community composition and network topology. The healthy fruit peels maintained a “functionally balanced” nutritional strategy for resource acquisition and host defense, hosting fungal communities with a diverse trophic mode (pathotroph-saprotroph-symbiotroph). This multifaceted functional repertoire of the fungal assemblages may contribute to nutrient cycling and systemic disease resistance in the fruit peel, consistent with reports that multifunctional fungal guilds enhance plant immunity and ecosystem stability in fruit and leaf microbiomes [15]. However, after disease onset, fungal functional traits clearly diverged. Specifically, plant pathogen and endophyte-plant pathogen guilds showed higher relative abundance in GD, which is consistent with the predicted presence of plant-pathogenic fungi. In contrast, the undefined saprotroph and wood saprotroph guilds showed higher relative abundance in RD. This pattern may reflect the potential for saprotrophic fungal proliferation in necrotic tissue, though direct evidence linking the observed guild shifts to tissue necrosis or nutrient availability was not obtained in this study. The microbial decomposition of dead host tissues may indirectly facilitate pathogen persistence by recycling nutrients and creating a favorable microenvironment for secondary colonizers [38]. Besides, a concurrent decline in fungal parasite-undefined saprotroph guilds was observed consistently across both diseased phenotypes (GD and RD), suggesting that pathogen invasion might disrupt native functional equilibrium and reduce community functional redundancy of the endophytic fungal assemblage, impairing the ability of these communities to withstand secondary infections or environmental perturbations. This shift in functional composition was evident in both immature (green-peel) and mature (red-peel) diseased fruits, which may be associated with disease-related changes in fungal community structure. However, whether this represents a genuine loss of functional resilience, a compositional effect of adding pathogen-associated taxa, or a combination of both, remains to be determined. Future studies employing absolute quantification and functional assays are needed to confirm whether the observed guild shifts correspond to biologically relevant changes in microbial defense capacity.

Collectively, these findings highlight that canker may represent an ecological process involving fungal community-wide restructuring from stochastic assembly to deterministic selection and from cooperative networks to fragmented or pathogen-dominated topologies, potentially challenging the conventional “single pathogen” paradigm of fruit disease. Besides, the identification of health-associated indicator taxa, such as *Cladosporium* and *Cryptococcus*, provides candidate biocontrol agents or microbial biomarkers for early disease detection. *Cladosporium* species are well-documented antagonists against phytopathogens through competitive exclusion and antibiotic production, while *Cryptococcus* yeasts are recognized as beneficial endophytes that enhance host immunity and nutrient acquisition [39,40]. The dominance of these taxa in healthy fruits and their significant reduction in diseased tissues suggest that preserving or augmenting these beneficial fungal populations could enhance the fruit’s resilience to canker disease. Meanwhile, the deterministic assembly observed in diseased immature fruits suggests that pre-harvest early intervention targeting niche modification of the fruit peel microenvironment, rather than pathogen eradication alone, may restore stochastic processes and ecological resilience, thereby re-establishing the protective microbial networks and enhancing resistance to secondary infections. Understanding these microbiome-disease dynamics offers a framework for microbiome-targeted disease management in tropical fruit crops, emphasizing the preservation of community stochasticity and functional guild diversity.

However, several methodological limitations of the present study should be acknowledged. First, the ITS1 amplicon sequencing approach has inherent biases. ITS1 primers preferentially amplify Ascomycota and Basidiomycota, whereas fungi with large ITS introns and non-Dikarya lineages (e.g., Chytridiomycota, Mucoromycota, and Glomeromycota) may be systematically underrepresented. Furthermore, genus-level identification represents the practical ceiling for reliable ITS-based environmental taxonomy; 152 ASVs (12.8% of the total ASV pool, accounting for 1.30% of sequences) remained unclassified at the phylum level, and 27.5% of ASVs were not assigned to the genus level. Second, all abundance data reported here are relative, and the observed shifts in community composition may be partially influenced by compositional effects. When disease-associated taxa colonize the fruit peel, the proportional representation of resident taxa may decrease even if their absolute abundance remains unchanged. Consequently, the observed “higher” or “lower” relative abundances do not necessarily reflect absolute changes in fungal biomass or cell numbers. Third, the functional assignments were generated using the FUNGuild database, which relies on genus-level taxonomy. Many fungal genera contain species with multiple trophic modes (e.g., endophytic, saprotrophic, and pathogenic), and the functional roles of poorly characterized taxa remain unknown. Of the 1185 ASVs, 889 (75.0%) were successfully assigned to a functional guild, collectively accounting for 97.4% of the total sequence abundance; the remaining 296 ASVs (25.0% of ASVs, but only 2.6% of sequences) were either unclassified or designated as unknown saprotrophs. Fourth, an ASV-based approach was employed rather than traditional OTU clustering. While ASVs offer higher resolution and reproducibility, they may inflate richness estimates due to intra-genomic ITS variation among fungi [27]. However, the major ecological patterns identified—including the stronger effect of ripening than disease status, the stage-dependent shift from stochastic to deterministic assembly, and the divergent co-occurrence network topologies—were consistent across both ASV and genus-level taxonomic resolutions. Future studies could benefit from complementary validation using OTU-based species-hypothesis thresholds previously proposed for fungal ITS data [24,25,26]. Finally, the study was conducted at a single orchard during one growing season, and the cross-sectional design precludes inference of temporal dynamics. The community assembly and co-occurrence patterns reported here are therefore correlative; the extent to which the observed disease-associated community shifts reflect genuine biological processes versus methodological artifacts cannot be definitively resolved without absolute-quantification methods and manipulative experiments.

## 5. Conclusions

The present study revealed that fruit ripening and canker onset collectively shape fungal communities in pitaya fruit peel. Ripening reduced fungal diversity and restructured community composition, while early pathogen invasion imposed strong selective pressure, shifting fungal community assembly from stochastic to deterministic processes, particularly in immature fruits. These changes in the fungal communities of both immature (green) and mature (red) fruit peels resulted in the breakdown of cooperative fungal network structures during disease progression. The immature healthy fruits displayed loose, modular fungal networks that were vulnerable to pathogen invasion, whereas mature healthy fruits maintained dense, interconnected fungal networks. Moreover, disease disrupted the cooperative fungal network structures in both developmental stages: immature infected fruits exhibited a transitional network topology with increased connectivity but reduced modularity, whereas mature infected fruits displayed a fragmented network architecture with reduced functional redundancy. These findings provide a theoretical basis for understanding microbiome-mediated disease resistance and for developing precision agriculture strategies tailored to specific fruit developmental stages. Nevertheless, future studies using metagenomics, metabolomics, and gene expression analysis will help clarify the mechanisms underlying changes in community assembly and network topology within the fruit peel microbiome, identifying specific fungal species as biomarkers or biocontrol agents for sustainable pitaya production.

## Figures and Tables

**Table 1:** PERMANOVA results showing the percentage of variation in fruit-associated fungal community structure explained by ripening stage and disease status.

Factors	Sums of Squares	Mean Squares	F. Models	*R* ^2^	*P*r (>F)
Ripeness	1.1840	1.1840	7.8138	0.2066	<0.001
Disease	0.7758	0.7758	4.6981	0.1354	<0.001
Ripeness × disease	2.6928	0.8976	8.2750	0.1280	<0.001
Full model (Total)	–	–	–	0.4700	–

**Table 2:** Topological parameters of the GH, GD, RH, and RD networks.

Parameters	GH *	GD *	RH *	RD *
No. of nodes	48	48	48	47
No. of edges	79	95	118	87
No. of positive edges/proportion (%)	50/63.29	84/88.42	97/82.20	71/81.61
No. of negative edges/proportion (%)	29/36.71	11/11.58	21/17.80	16/18.39
Clustering coefficient	0.387	0.381	0.523	0.398
Avg. degree	3.292	3.958	4.917	3.702
Betweenness centrality	0.064	0.083	0.026	0.048
Closeness centrality	0.195	0.219	0.219	0.243
Modularity	0.673	0.534	0.565	0.613
Number of Communities	10	7	6	6
Network density	0.07	0.084	0.105	0.08
Average path length	4.509	4.397	2.911	3.558

* GH: immature and healthy; * GD: immature and diseased; * RH: mature and healthy; * RD: mature and diseased.

## Data Availability

The data presented in this study have been deposited in the NCBI Sequence Read Archive (SRA) under BioProject accession number PRJNA1441134 (https://www.ncbi.nlm.nih.gov/sra/PRJNA1441134, accessed on 26 March 2026). The data will be released on 31 December 2026.

## Supplementary Materials

The following supporting information can be downloaded at: https://www.mdpi.com/article/10.3390/microorganisms14071441/s1. Figure S1: Venn diagram showing the number of fruit-associated fungal ASVs among the four groups.

## Abbreviations

The following abbreviations are used in this manuscript:

ANOSIM	Analysis of similarities
ASV	amplicon sequence variant
dNTP	deoxynucleoside triphosphate
DNA	deoxyribonucleic acid
DPF	days post-flowering
FDR	False Discovery Rate
GD	immature (green-peeled) and diseased
GH	immature (green-peeled) and healthy
ITS	internal transcribed spacer
LDA	Linear Discriminant Analysis
LEfSe	Linear Discriminant Analysis Effect Size
NST	normalized stochasticity ratio
PCoA	Principal Coordinates Analysis
PCR	polymerase chain reaction
PERMANOVA	permutational multivariate analysis of variance
RD	mature (red-peeled) and diseased
RH	mature (red-peeled) and healthy
sp.	species
spp.	species pluralis
USD	United States Dollar
vs.	versus
