# Supplementary material for: Fungal Communities Within Pitaya Fruit Peel Shift During Ripening and Early Canker Onset
Source: Microorganisms. 2026 Jun 30;14(7):1441. doi: 10.3390/microorganisms14071441 (PMC13413601; doi:10.3390/microorganisms14071441)
Supplement: Supplementary file 1 [file microorganisms-14-01441-s001.zip › microorganisms-4373225-supplementary.pdf]

Supplementary Materials for

Fungal Communities within Pitaya Fruit Peel Shift during Ripening and Early Canker Onset

Ziting Yao[1*], Yanling Zhao[1,2], Lianke Zhu[1,2], Guining Zhu[1], Chengwu Zou[2*]

[1] Plant Protection Research Institute, Guangxi Academy of Agricultural Sciences / Key Laboratory of Green Prevention and Control on Fruits and Vegetables in South China, Ministry of Agriculture and Rural Affairs / Guangxi Key Laboratoryof Biology for Crop Diseases and Insect Pests, Nanning, China
[2] College of Agriculture / National Demonstration Center for Experimental Plant Science Education, Guangxi University, Nanning, China

*Corresponding authors: youziting@163.com; zouchengwu@gxu.edu.cn

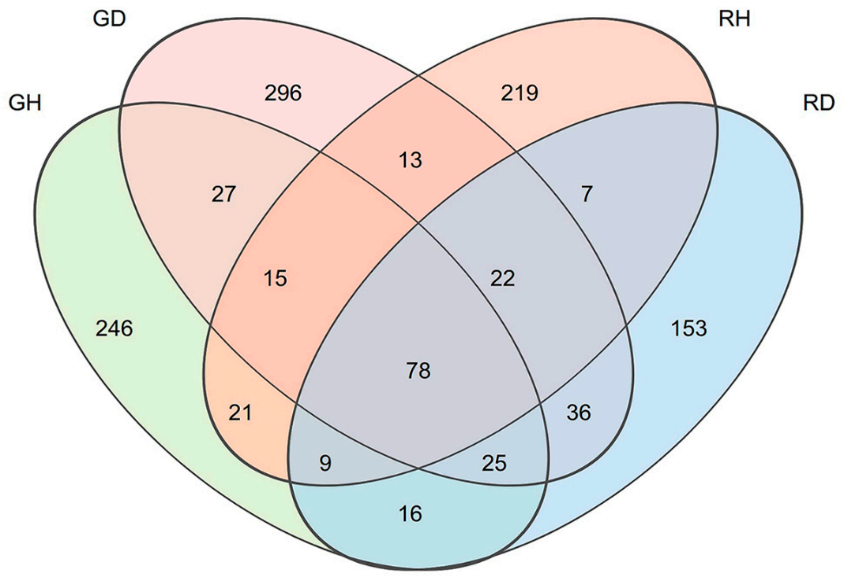

Figure S1. Venn diagram showing the number of fruit-associated fungal ASVs among the four groups.

GH: immature and healthy; GD: immature and diseased; RH: mature and healthy; RD: mature and diseased.
